# Phase II clinical trial of adoptive cell therapy for patients with metastatic melanoma with autologous tumor-infiltrating lymphocytes and low-dose interleukin-2

**DOI:** 10.1007/s00262-019-02307-x

**Published:** 2019-02-11

**Authors:** Linh T. Nguyen, Samuel D. Saibil, Valentin Sotov, Michael X. Le, Leila Khoja, Danny Ghazarian, Luisa Bonilla, Habeeb Majeed, David Hogg, Anthony M. Joshua, Michael Crump, Norman Franke, Anna Spreafico, Aaron Hansen, Ayman Al-Habeeb, Wey Leong, Alexandra Easson, Michael Reedijk, David P. Goldstein, David McCready, Kazuhiro Yasufuku, Thomas Waddell, Marcelo Cypel, Andrew Pierre, Bianzheng Zhang, Sarah Boross-Harmer, Jane Cipollone, Megan Nelles, Elizabeth Scheid, Michael Fyrsta, Charlotte S. Lo, Jessica Nie, Jennifer Y. Yam, Pei Hua Yen, Diana Gray, Vinicius Motta, Alisha R. Elford, Stephanie DeLuca, Lisa Wang, Stephanie Effendi, Ragitha Ellenchery, Naoto Hirano, Pamela S. Ohashi, Marcus O. Butler

**Affiliations:** 1 Tumor Immunotherapy Program, Princess Margaret Cancer Centre, Toronto, Canada; 2 Department of Medical Oncology and Hematology, Princess Margaret Cancer Centre, Toronto, Canada; 3 Department of Laboratory Medicine, University Health Network, Toronto, Canada; 4 Kinghorn Cancer Centre, St. Vincent’s Hospital, Sydney, Australia; 5 Department of Surgical Oncology, Princess Margaret Cancer Centre, Toronto, Canada; 6 Department of Otolaryngology, Head and Neck Surgery, Princess Margaret Cancer Centre, Toronto, Canada; 7 Department of Pharmacy, Princess Margaret Cancer Centre, Toronto, Canada; 8 Drug Development Program, Princess Margaret Cancer Centre, Toronto, Canada; 9 Department of Immunology, University of Toronto, Toronto, Canada; 10 University Health Network, Princess Margaret Cancer Centre, 9-622, 610 University Avenue, Toronto, ON M5G 2M9 Canada

**Keywords:** Tumor-infiltrating lymphocytes, Adoptive cell therapy, Metastatic melanoma, Interleukin-2, Clinical trial

## Abstract

Adoptive cell therapy using autologous tumor-infiltrating lymphocytes (TIL) has shown significant clinical benefit, but is limited by toxicities due to a requirement for post-infusion interleukin-2 (IL-2), for which high dose is standard. To assess a modified TIL protocol using lower dose IL-2, we performed a single institution phase II protocol in unresectable, metastatic melanoma. The primary endpoint was response rate. Secondary endpoints were safety and assessment of immune correlates following TIL infusion. Twelve metastatic melanoma patients were treated with non-myeloablative lymphodepleting chemotherapy, TIL, and low-dose subcutaneous IL-2 (125,000 IU/kg/day, maximum 9–10 doses over 2 weeks). All but one patient had previously progressed after treatment with immune checkpoint inhibitors. No unexpected adverse events were observed, and patients received an average of 6.8 doses of IL-2. By RECIST v1.1, two patients experienced a partial response, one patient had an unconfirmed partial response, and six had stable disease. Biomarker assessment confirmed an increase in IL-15 levels following lymphodepleting chemotherapy as expected and a lack of peripheral regulatory T-cell expansion following protocol treatment. Interrogation of the TIL infusion product and monitoring of the peripheral blood following infusion suggested engraftment of TIL. In one responding patient, a population of T cells expressing a T-cell receptor Vβ chain that was dominant in the infusion product was present at a high percentage in peripheral blood more than 2 years after TIL infusion. This study shows that this protocol of low-dose IL-2 following adoptive cell transfer of TIL is feasible and clinically active. (ClinicalTrials.gov identifier NCT01883323.)

**Electronic supplementary material:**

The online version of this article (10.1007/s00262-019-02307-x) contains supplementary material, which is available to authorized users.

## Introduction

Adoptive cell therapy (ACT) using autologous tumor-infiltrating lymphocytes (TIL) has demonstrated tremendous potential for treatment of advanced tumors, particularly melanoma (Reviewed in [1]). Current ACT protocols incorporate the use of preparative non-myeloablative lymphodepleting chemotherapy regimens before the infusion of the ex vivo expanded autologous TIL. Subsequent to the TIL infusion, patients are treated with high-dose interleukin-2 (IL-2). Using this approach, objective response rates ranging from 38 to 50% have been reported in patients with metastatic melanoma, with durable, complete tumor regression observed in up to 20% of patients [2–6]. These data indicate that TIL therapy offers the potential for durable clinical benefit, even in patients with few treatment options.

The tradeoff for these encouraging results is that current treatment protocols of TIL therapy result in a high rate of grade 3 or 4 toxicities. The preparative chemotherapy has been demonstrated to increase the efficacy of therapy, in part by increasing the availability of homeostatic cytokines, such as IL-15, for the infused TIL as well as by depleting T-regulatory cells (Tregs) [7–10]. Unfortunately, the preparative chemotherapy also commonly results in anemia and thrombocytopenia requiring transfusions and sustained neutropenia requiring growth factor support [7]. The IL-2 given post-TIL infusion, to support survival and expansion of the transferred TIL, has typically been administered as high-dose, intravenous IL-2 at a dose of 720,000 IU/kg every 8 h as tolerated for a maximum of 5 days [2–5]. High-dose IL-2 is also associated with significant toxicity including neurological symptoms and a systemic capillary leak syndrome that can result in significant hypotension, renal failure, and pulmonary edema requiring intensive care unit (ICU) care [11]. Attempts have been made to alter the current TIL protocols to limit this toxicity via modifications of the IL-2 dosing regimen post-TIL infusion [12–14]. A pilot study of subcutaneously administered IL-2 at a dose of 2 MIU/day for 14 days demonstrated durable clinical responses in two of the six patients [13]. In addition, a larger trial of 25 patients demonstrated an objective response rate of 42% utilizing a “decrescendo” regimen of continuous, intravenous IL-2 at a dose of 18 MIU/m^2^ over 6, 12 and then 24 h followed by 4.5 MIU/m^2^ over 24 h for 3 days [14]. These data indicated that high-dose IL-2 is not an absolute requirement to derive clinical benefit from TIL therapy and that further investigations into modified IL-2 dosing regimens are warranted. Here, we report the results of a phase II study of 12 patients with metastatic melanoma treated with a modified ACT protocol utilizing autologous TIL with preconditioning chemotherapy followed by the administration of subcutaneous IL-2 administered at a low dose of 125,000 IU/kg/day over 12 days.

## Materials and methods

### Patients

All patients underwent informed consent to participate in this study. Patients 18 years or older with unresectable stage III or stage IV melanoma, per the 7th edition of the AJCC staging [15], were eligible for enrollment. Other requirements were an Eastern Cooperative Oncology Group (ECOG) performance status of 0 or 1 and a life expectancy of greater than 3 months from the date of consent to TIL treatment. Patients with brain metastases were eligible provided that they had three or fewer asymptomatic lesions each measuring less than or equal to 1 cm. Alternatively, patients with brain metastases not meeting these criteria were eligible if they had definitive treatment with surgery and/or radiation at least 30 days prior to the first dose of lymphodepleting chemotherapy. Key exclusion criteria were active chronic infections, continuing requirement for systemic corticosteroid treatment as well as significant medical comorbidities including active cardiac illness or pulmonary dysfunction.

### Study design

The primary endpoint of this study was clinical efficacy as defined by response according to the Response Evaluation Criteria in Solid Tumors (RECIST) guideline (version 1.1) [16]. Response was also assessed using the Immune-related Response Criteria (irRC) [17]. Secondary endpoints were to evaluate the safety of this low-dose IL-2 treatment protocol and to evaluate the immune status of patients following treatment. Toxicity was assessed using the Common Terminology Criteria for Adverse Events (CTCAE) version 4.0.

All patients received a non-myeloablative, lymphodepleting chemotherapy regimen consisting of cyclophosphamide (60 mg/kg/day) for 2 days (days − 5 and − 4) as well as fludarabine (25 mg/m^2^/day) for 5 days (days − 5 to − 1) as an inpatient before TIL infusion. At least 1 week prior to the initiation of this preparative chemotherapy, all patients also underwent mobilization with filgrastim and leukapheresis to cryopreserve hematopoietic stem cells. TIL were infused on day 0 of the protocol, and on the same day, subcutaneous IL-2 was initiated at a dose of 125,000 IU/kg/day. IL-2 was administered daily as an inpatient over a 12-day period with a 2-day break after the first 4–5 doses of IL-2 (maximum 9–10 doses). The regimen was based on the low-dose regimen described by Yang et al. [18], and modified with the aim of further reducing toxicities. All patients were treated with daily filgrastim injections as well as ciprofloxacin and cloxacillin, while absolute neutrophil count (ANC) was less than 1.0 × 10^9^/L. Prophylaxis with acyclovir and fluconazole was initiated at the end of chemotherapy. Prophylactic trimethoprim and sulfamethoxazole were initiated upon hospitalization and continued until absolute CD4+ count was above 0.2 × 10^9^/L and for at least 6 months after chemotherapy. Platelet and red blood cell transfusions were given as needed to maintain platelets greater than 10 × 10^9^/L and hemoglobin greater than 80 g/L.

### TIL culturing

The TIL manufacturing procedure was adapted from Dudley et al. [19]. Melanoma tissue was processed by cutting into ~ 1 mm^3^ fragments. Tissue fragments were either plated directly into 24-well plates or enzymatically dissociated in medium comprised of Iscove’s Modified Dulbecco’s Medium (IMDM) (Lonza) containing Collagenase (Sigma) and Pulmozyme (Roche) and then plated in 24-well plates at a concentration of 1.0 × 10^6^ cells/well. Cells were cultured in complete medium comprised of: IMDM (Lonza), 10% healthy donor plasma (prepared in-house, as described in Nguyen et al. [20]), HEPES (Lonza), penicillin/streptomycin (Lonza) (omitted for patients with penicillin allergy), gentamycin (Lonza), 2-mercaptoethanol (Invitrogen), l-glutamine (Lonza), and 6,000 IU/mL IL-2 (Proleukin, Novartis). Cells were maintained at a concentration of approximately 1.0 × 10^6^ cells/mL and expanded for a maximum of 28 days prior to cryopreservation and QC testing. In general, four independent, bulk TIL cultures were established from each patient specimen.

### Rapid expansion protocol (REP)

TIL from two independent bulk TIL cultures were thawed, rested, and seeded in parallel in Rapid Expansion Protocols (REPs) with 30 ng/mL OKT3 (GMP Grade) (Miltenyi Biotec), irradiated (50 Gy) allogeneic PBMC feeder cells (1:100 TIL:PBMC), and 600 IU/mL of IL-2 in “50/50” media containing 50% complete medium (as described above) prepared using human serum AB+ (Gemini Bio Products) and 50% AIM V media (Invitrogen). Fungizone (Lonza) was added on day 5 onwards. Note that a lower concentration of IL-2 was used in the REP compared to the protocol developed by Dr. Steven Rosenberg (Surgery Branch, National Cancer Institute). The REP was performed in G-Rex100 flasks (Wilson Wolf) for all patients except for the first patient treated. For Patient 1, the REP was initiated in T175 flasks and then transferred to 3L culture bags (Fenwal) on day 7. TIL were harvested on day 14 of the REP for all patients except Patient 5, whose TIL were harvested on day 15 due to a change in the clinical schedule. The final product was prepared in infusion media comprised of 2.5% human serum albumin, 300 IU/mL IL-2 in 0.9% saline (Baxter) and transferred to a transfer pack (Fenwal) for infusion.

For TIL manufacturing and QC, percent CD3^+^ was assessed by flow cytometry (clone UCHT1). Two independent bulk cultures of TIL were selected for the REP based on features such as enrichment for CD8^+^ cells and in vitro tumor reactivity; however, TIL cultures selected for the REP were not required to satisfy any criteria related to % CD8^+^ cells or tumor reactivity.

### Tumor reactivity assays

The tumor reactivity of pre-REP TIL was assayed in vitro when possible. IFN-γ production was assessed by co-culturing TIL with target cells overnight and assaying the supernatant by ELISA (eBioscience/Thermo). Positive IFN-γ activity was defined as secretion of > 200 pg/mL IFN-γ above baseline (TIL alone) with at least a 50% decrease in IFN-γ secretion in the presence of anti-HLA Class I antibody (clone W6/32). Target cells were cryopreserved, enzyme-dissociated, autologous tumor in all cases except for Patient 9, where MART1/Melan-A-peptide-loaded HLA-A*02:01 + lymphoblastoid (T2) or HLA-A-matched or mismatched melanoma cell lines (526mel, 888mel, respectively; kind gifts from Dr. S. Rosenberg) were used as surrogate targets. Cytotoxicity (CTL) assays were performed by standard chromium (^51^Cr) release assay. TIL were considered to be tumor reactive if all the following criteria were satisfied: (1) > 10% specific lysis at either one of a 60:1 or 20:1 effector:target ratio; (2) an overall decrease in specific lysis with each decrease in effector:target ratio; and (3) a > 25% reduction in specific lysis in the presence of anti-HLA Class I antibody (clone W6/32) compared to specific lysis in the absence of W6/32 at an effector:target ratio of either 60:1 or 20:1.

### Microbead immunoassay

Concentrations of cytokines were measured in serum using the Bio-Plex Pro Human Cytokine 27-plex Assay (Bio-Rad) per manufacturer’s instructions. Serum obtained from 10 healthy donors was also tested to establish a normal range for each cytokine. Samples were run and analyzed using the Bio-Plex MAGPIX multiplex reader.

### Flow cytometry analysis

Phenotypic analysis was performed on freshly isolated PBMC using the following monoclonal antibodies. From Fisher Scientific: PD-1 (J105), CD4 (RPA-T4), FoxP3 (236A/E7), CD127 (eBioRDR5), CD25 (4E3); from BioLegend: CD8 (SK1), CD3 (UCHT1), CD14 (M5E2), and CD19 (H1B19). The TCR Vβ repertoire was analyzed using the IOTest Beta Mark Kit (Beckman-Coulter). Staining for MART-1/Melan-A [26–35 (27L); ELAGIGILTV] specific T cells was performed using APC-bound pentamers (ProImmune). Intracellular staining (FoxP3) was performed after applying fixation/permeabilization buffer (Fisher Scientific). All samples were run on a Canto II flow cytometer and analyzed using FlowJo Ver. 9.5.8. Dominant TCR Vβ populations were identified based on any Vβ chain whose frequency was considered to be a statistical outlier in the repertoire of the 24 Vβ chains that were analyzed. An outlier test was used to define a Vβ as dominant if its frequency was at least three interquartile distances away from the third quartile of all the Vβ chains analyzed.

### Statistics

A two-stage Simon design for a phase II trial was followed: A response rate of 5% or lower was considered too low to be of interest (p0 = 0.05), while if the treatment had a response rate of 30% or more, it was considered to be effective (pA = 0.3). A total of 12 patients were required to be accrued to this study with an overall probability to reject an effective treatment of 0.198 and an overall probability to accept an ineffective treatment of 0.084. Accrual was conducted in stages. During the first stage, five patients were accrued. If there were no responders, then the study would have stopped with the conclusion that treatment was ineffective. If there was at least one responder, then the study would continue to the second stage and accrue seven more patients to a total of 12. If by the end of the study, there were one or less responders in the total of 12 patients, the treatment would be considered ineffective. If there were two or more, then the treatment would be considered effective.

## Results

### Patient and TIL characteristics

Between October 2013 and December 2017, 12 patients with metastatic melanoma were treated on protocol. Within this cohort, one patient had a melanoma of ocular origin, two had mucosal melanomas, and the remaining had cutaneous melanoma. All of the patients except one had M1c disease, as per the American Joint Committee on Cancer (AJCC) 7th edition classification, with the remaining patient having M1b disease. Four had previously-treated brain metastases before enrollment in the trial. All patients had been heavily pretreated for their metastatic disease, with the exception of one patient who did not receive prior therapy. Ten of the 12 patients had received prior treatment with ipilimumab and nine had received prior programmed death-1 (PD-1) blockade with either nivolumab or pembrolizumab. Two patients also had received combination immunotherapy with combined ipilimumab and nivolumab. In addition, eight patients had been treated with chemotherapy. The baseline characteristics of the patients are summarized in Table 1.

**Table 1:** Patient characteristics

Patients	Sex	Age	Histology	BRAF status	M stage^a^	Disease sites	Previous treatment
1	M	43	Cutaneous	WT	M1b	LN, SC, lung	None
2	M	64	Cutaneous	WT	M1c	LN, lung, liver, adrenal	Ipi/Nivo, Carbo-tax
3	F	35	Cutaneous	WT	M1c	Lung, Sp, peritoneum, bowel	Carbo-tax, Ipi
4	M	48	Cutaneous	V600E	M1c	Brain, LN, lung, Sp, kidney, gallbladder, psoas	Dabrafenib/Trametinib, Ipi, Pembro
5	F	40	Mucosal	WT	M1c	SC, lung, liver, kidney, retroperitoneum	Carbo-tax, Ipi, DTIC
6	F	49	Mucosal	WT	M1c	LN, lung, pleura, uterus, bone	Ipi, Pembro, Carbo-tax
7	M	49	Cutaneous	WT	M1c	Brain, LN, SC, lung, pleura, chest wall, liver, Sp, small bowel	Ipi, Pembro
8	M	35	Cutaneous	WT	M1c	LN, SC, lung, Sp, kidney, bone, ureter, pancreas	DTIC, Ipi, Pembro, Carbo-tax
9	F	34	Cutaneous	WT	M1c	LN, SC, lung, peritoneum, liver, kidney, breast	DTIC, Ipi, Pembro, IL-2 (injections)
10	M	61	Cutaneous	WT	M1c	Brain, LN, lung, kidney, pleura, perinephric space	Ipi/Nivo, Pembro
11	M	42	Uveal	WT	M1c	LN, SC, lung, peritoneum, liver, kidney, pleura	DTIC/Selumetinib, Ipi/Nivo, Pembro
12	M	61	Cutaneous	WT	M1c	Brain, LN, SC, lung, peritoneum, liver, pericardium, adrenal gland	Nivo, anti-PD-1/anti-GITR, Carbo-tax

*WT* wild type, *LN* lymph nodes, *SC* subcutaneous, *Sp* spleen, *Ipi* ipilimumab, *Nivo* nivolumab, *Carbo-tax* carboplatin + paclitaxel, *Pembro* pembrolizumab, *DTIC* dacarbazine

^a^Based upon AJCC 7th Edition

The method for in vitro TIL expansion was adapted from Dr. Steven Rosenberg (NCI) [19]. Of note, the concentration of IL-2 that we used during the second stage of expansion (Rapid Expansion Protocol; REP) (600 IU/mL) was lower than that used by the Rosenberg group and commonly used in other TIL protocols (3000 IU/mL). The characteristics of the TIL cultures and the infusion products are summarized in Table 2. TIL were harvested from sites other than lymph nodes in 8 of 12 patients. The average fold expansion during the REP was 2619 fold (range 1159–4700). This expansion allowed an average of 1.12 × 10^11^ cells to be infused for treatment (range 5.5 × 10^10^–1.6 × 10^11^). The percentage of CD8^+^ versus CD4^+^ T cells in each infusion product was determined. Eight of the 12 infusion products (Patients 1, 4, 6–8, and 10–12) had a CD8^+^ dominant phenotype with greater than 60% CD8^+^ T cells. Patient 5 received a CD4^+^ dominant TIL product, with greater than 60% being CD4^+^ lymphocytes. The remaining three patients (Patients 2, 3, and 9) comprised a third group, receiving more balanced infusion products in which neither T-cell population exceeded 60%.

**Table 2:** Pre-REP TIL and TIL infusion product characteristics

Patients	Tumor specimen from LN?	Days in pre-REP culture (per TIL culture)	REP fold-expansion	Number of cells infused	Infusion product	
					%CD8	%CD4
1	N	21, 22	1159	5.50 × 10^10^	82.0	5.4
2	Y	14, 14	1700	8.60 × 10^10^	44.8	54.1
3	Y	16, 17	2040	1.00 × 10^11^	48.2	39.4
4	Y	18, 18	2640	1.32 × 10^11^	85.3	10.5
5	N	20, 20	1524	8.00 × 10^10^	30.2	69.3
6	Y	14, 17	2750	1.06 × 10^11^	80.2	15.5
7	N	14, 14	2917	1.46 × 10^11^	93.0	6.2
8	N	17, 17	4700	1.60 × 10^11^	62.3	35.2
9	N	16, 16	2560	8.00 × 10^10^	47.9	39.5
10	N	16, 16	4452	1.56 × 10^11^	96.1	3.7
11	N	22, 26	2017	9.84 × 10^10^	81.2	18.2
12	N	18, 18	2972	1.46 × 10^11^	73.3	21.4

*LN* lymph node, *TIL* tumor-infiltrating lymphocyte, *REP* Rapid Expansion Protocol

### Treatment-related toxicity

The incidence and severity of each treatment-related adverse event observed during the trial are summarized in Table 3. There were no grade 5 adverse events related to study therapy. All of the patients experienced grade 3–4 hematological toxicities as expected with lymphodepleting chemotherapy. In ten patients, these toxicities were reversible with blood and platelet transfusions and G-CSF growth factor support. One patient, however, experienced refractory pancytopenia with marrow aplasia found on bone marrow biopsy. Infusion of the patient’s preserved hematopoietic stem cells restored hematopoiesis and the patient’s cell counts subsequently recovered. A second patient also received stem cells on day 13 for delayed recovery of cell counts in the setting of extensive melanoma bone metastasis and marrow involvement. This patient’s cell counts recovered with no infectious or bleeding complications.

**Table 3:** Adverse events related to treatment

Toxicities	Number (maximum grade)			
	Grade 1	Grade 2	Grade 3	Grade 4
Hematologic				
Neutropenia			5	7
Thrombocytopenia			6	5
Leukopenia			11	
Lymphopenia		1	2	4
Anemia	1		10	
Bone marrow aplasia/delayed engraftment			2	
Febrile neutropenia			7	
Gastrointestinal				
LFT increase	7	1		
Bilirubin	1	2		
Nausea/vomiting	6	9		
Diarrhea	4	6		
Pain	4	3		
Mucositis	1	1		
Constipation	1	1		
Anorexia	3	2		
Blood in stool and melena		2		
Renal				
Electrolyte imbalance	7	5	3	
AKI creatinine	6			
Hematuria	4	2		
Urine output decreased			3	
Urinary retention			2	
Proteinuria	1			
Neurologic				
Fatigue	3	4	1	
Headache	2	3		
Hallucinations	1			
Insomnia	2			
Paresthesia	1			
Drowsiness and mental status change	2			
Musculoskeletal				
Pain	3	1		
CPK increased	1			
Arthralgia		1		
Vascular leak syndrome				
Edema, weight gain	10	3	1	
Pulmonary edema		2		
Hypotension	1	1		
Dyspnea respiratory distress	3	4	1	
Hypoxia		2	3	
Capillary leak syndrome		3		
Pleural effusion	1			
Pericardial effusion		1		
Dermatologic				
Rash	4	2	1	
Pruritus	1			
Alopecia	2			
Skin hypopigmentation (Vitiligo)	2			
Infections				
Fever	4	5		
EBV infection	1			
RSV viral infection	1			
Pleural infection		1		
Upper respiratory infection		1		

*LFT* liver function test, *AKI* acute kidney injury, *CPK* creatine phosphokinase test, *EBV* Epstein–Barr virus, *RSV* respiratory syncytial virus

Febrile neutropenia was also a commonly observed toxicity in these patients after lymphodepletion. In six patients, no infectious source for the fever was ever identified. Conversely, the patient with aplastic bone marrow also developed multiple infectious complications including a positive nasopharyngeal swab for respiratory syncytial virus for which oral ribavirin therapy was initiated and *Streptococcus viridans* and *Klebsiella oxytoca* bacteremia that was treated with antibiotics.

Low-dose IL-2 was reasonably well tolerated with patients receiving an average of 6.8 doses (range from 2 to 9 doses). The majority of the toxicities directly attributable to IL-2 were grade 1 or 2 and were those relating to vascular leak syndrome, including peripheral edema, pulmonary edema, hypotension and increased creatinine or decreased urine output, as well as fever, fatigue and neurological symptoms. In most patients, these symptoms could be managed with supportive measures and by delaying or omitting subsequent IL-2 injections. Eight of the 12 patients were able to receive at least seven doses of the targeted nine doses of IL-2 and five patients received all nine doses. Three patients only received four injections. One of these patients developed a grade 3 pruritic rash that was determined to be related to trimethoprim and sulfamethoxazole, and as a precaution, further IL-2 injections were held. Of the other two patients who only received four doses of IL-2, one developed grade 3 edema, and the other developed grade 2 edema and grade 1 renal failure that resolved with the discontinuation of IL-2. Patient 9, who had progressive melanoma metastases, received only two doses of IL-2 due to IL-2-induced fluid retention. Nine days following the last dose of IL-2, this patient went on to develop pulmonary infiltrates, hypoxia, and respiratory distress requiring temporary mechanical ventilation. This episode was attributed to an immune reconstitution syndrome rather than the previous IL-2. Overall, most patients were able to receive six or more doses of low-dose IL-2 with acceptable toxicities that could be managed in the non-ICU setting.

The other grade 3 toxicities observed were either related to the chemotherapy, such as electrolyte imbalances, or to the infusion of TIL. Two patients experienced hypoxic episodes immediately following infusion of TIL. In both cases, these episodes were transient and resolved with supportive treatment, but did result in a delay in starting IL-2.

### Clinical efficacy

When clinical response was assessed using RECIST v1.1 criteria, two patients (Patients 1 and 8) achieved a partial response (PR) and one patient (Patient 7) had an unconfirmed partial response (PRu). The rest of the patients had either stable disease (SD) lasting less than 6 months or progressive disease (PD) at the time of the first assessment (6 SD, 3 PD). Per RECIST v1.1 criteria, the estimated median progression free survival was 5.1 months (95%CI: 1.2–6.4 months). The estimated median overall survival was estimated to be 6.2 months (1.5 months—not reached), with five deaths being observed on trial (Supplementary Fig. 1). Applying the irRC criteria increased the number of confirmed PRs to three, as the patient with a PRu according to RECIST had continued decrease in the size of total lesion burden despite the presence of new liver lesions. This patient unfortunately had rapid progression of these liver lesions as well as disease elsewhere, including cutaneous lesions, shortly after having his PR confirmed. In the rest of the patients, we did not observe any evidence of late responses or pseudoprogression. A caveat, however, was that few patients had the requisite repeat imaging studies to confirm progression by irRC, because most patients had significant clinical progression that necessitated starting subsequent therapies before these studies could be obtained.

**Fig. 1:**
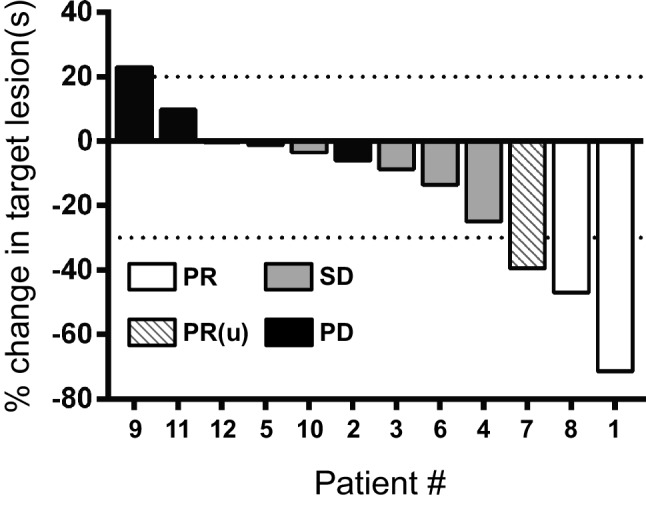
Waterfall plot. The best percentage change in the target lesion(s) is shown for each patient. The dotted lines show the threshold for progressive disease versus stable disease of target lesions (+ 20%) and partial response versus stable disease of target lesions (− 30%). The best response by RECIST v1.1 is shown for each patient. *PR* partial response, *PR(u)* unconfirmed partial response, *SD* stable disease, *PD* progressive disease

Despite only achieving clinical responses in 3 of the 12 patients, there was evidence of anti-tumor activity of the TIL in two additional patients. As depicted in the waterfall plot in Fig. 1, Patients 4 and 6 had tumor regression of greater than 10%; exhibiting 25%, and 14% reduction of target lesions, respectively. In all of these patients, the observed peak regression of target lesions was documented on the first radiographic assessment after treatment, with subsequent progression.

### Immune monitoring and TIL characteristics

**Fig. 2:**
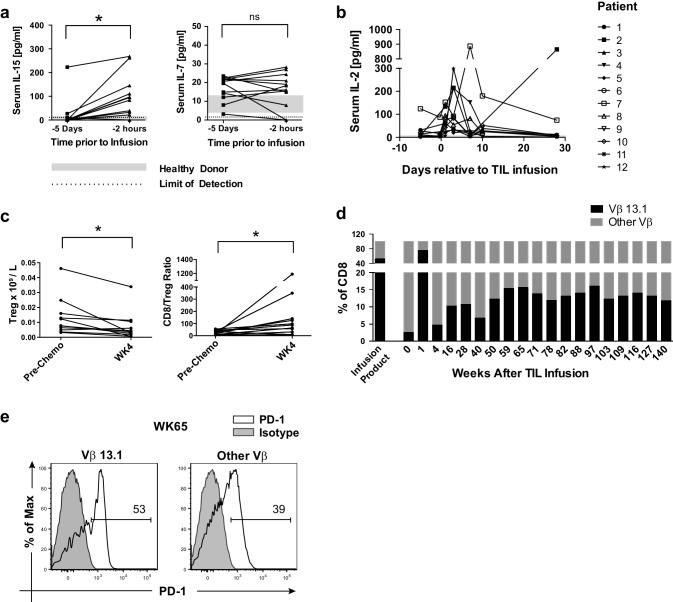
Immune monitoring. **a** Serum samples were collected from each patient before lymphodepleting chemotherapy (− 5 days) and 2 h before TIL infusion and assayed by multiplex assay for IL-15 and IL-7. Shaded areas represent the range of serum cytokine concentration in healthy donors (*n* = 10) **b** Serum samples were collected from each patient at the indicated time points and assayed for IL-2 using a multiplex assay. **c** Number of regulatory T cells per liter of blood as well as the ratio of CD8^+^ T cells/Tregs before chemotherapy and 4 weeks after TIL infusion was quantified. Tregs were defined as CD3^+^CD4^+^CD25^high^CD127^low^FoxP3^+^ cells. **d** For Patient 1, the percentage of Vβ13.1^+^ T cells in the CD8^+^ compartment in the TIL infusion product and in peripheral blood at the indicated time points was analyzed. **e** Peripheral blood of Patient 1 at 65 weeks after TIL infusion was analyzed for PD-1 expression, gated on CD3^+^ Vβ13.1^+^ T cells (left) or CD3^+^ Vβ13.1^−^ T cells (right)

Multiple immunological parameters were monitored throughout the protocol. The serum levels of the homeostatic cytokines IL-7 and IL-15 were assessed to evaluate the impact of lymphodepleting chemotherapy. We found a significant increase in serum levels of IL-15 following preparative lymphodepleting chemotherapy but no increase in the level of IL-7 **(**Fig. 2a**)**. These data are consistent with a previous study that found that the cyclophosphamide and fludarabine regimen increased serum IL-15 levels, but the addition of total body radiation was required to enhance serum IL-7 levels [10]. The observed increase in serum IL-15, however, indicated that lymphodepletion was effective and there was increased availability of this homeostatic cytokine for the infused TIL.

The serum level of IL-2 was also monitored. After TIL infusion, serum levels of IL-2 increased significantly (Fig. 2b). This increased IL-2 was most evident during the first 72 h following TIL infusion, during which time the patients were also being treated with subcutaneous IL-2. These data suggest that the exogenous IL-2 was given at a sufficient dose to alter IL-2 levels in the circulation. Alternatively, it is also possible that the infused TIL themselves were producing IL-2 and were partially responsible for the increase observed. Regardless of source, an increase in serum IL-2 levels was clearly observed following TIL infusion on this treatment protocol. Although thought to be beneficial for the expansion of the transferred TIL, exogenous IL-2 has also been implicated in the preferential expansion of peripheral CD4^+^ FoxP3^+^ regulatory T cells (Tregs) [21]. Accordingly, we monitored Tregs in the peripheral blood and observed the inverse: a decrease in the absolute number of Tregs and an increase in the ratio of CD8^+^ cells to Tregs and (Fig. 2c). Thus, it appeared that all patients had increased serum levels of IL-2 without peripheral expansion of Tregs.

Next, we characterized the expanded TIL to determine whether any characteristics were associated with clinical response. The anti-tumor reactivity of TIL was assayed by two different approaches. First, we tested HLA class I-dependent interferon-γ (IFN-γ) production as well as the class I-dependent cytolytic ability of the TIL cultures against autologous tumor (or for Patient 9, against HLA-A-matched tumor cells). We were able to test these in vitro functions in 10 of the 12 patients. However, we did not observe a discernable pattern to these results that was related to clinical response (Table 4). Amongst the responding patients, the TIL cultures from Patient 1 both produced IFN-γ in response to autologous tumor, while those from Patient 8 did not. These assays were not performed for Patient 7 (PRu). In addition, many of the TIL cultures from the non-responding patients produced IFN-γ and/or demonstrated cytolytic activity. Thus, in our cohort, these in vitro assays of anti-tumor activity were not predictive of response. Second, we determined the percentage of CD8^+^ T cells in the infusion product of each HLA-A*02:01-positive patient that recognized the tumor-associated antigen, Melanoma-associated antigen recognized by T cells (MART-1/Melan-A). Eight of the 12 patients were HLA-A*02:01-positive, and amongst those, the percentage of MART-1/Melan-A reactive cells in the infusion product was more than ninefold higher than in the baseline blood samples in six of the eight patients (Table 4). The two patients who had a confirmed PR did not show an enrichment of MART-1/Melan-A-reactive CD8^+^ T cells in the TIL infusion product. Thus, recognition of the HLA-A*02:01 MART-1/Melan-A epitope was not associated with clinical response. In the study reported by Radvanyi et al. [3], there was an association between the total number of CD8^+^ TIL infused and clinical response. We did not observe this association in our cohort, although interestingly, the three responding patients all had a dominance of CD8^+^ T cells in the infusion product (Table 2).

**Table 4:** Tumor reactivity of TIL

Patients	HLA-A	IFN-γ^a^	Cytotoxicity^a^	% MART-1 baseline PBMC^b^	%MART-1 infusion product^b^	%MART-1 Week 1 PBMC^b^	%MART-1 Week 4 PBMC^b^
1	0201/0201	2/2	1/2	0.03	0	0	0
2	0101/0301	2/2	2/2	N/A	N/A	N/A	N/A
3	0201/2403	0/2	2/2	0.30	2.92	1.68	0.42
4	0201/32	Not done	Not done	0.17	5.23	Not done	1.26
5	0201/2402	2/2	2/2	0.04	58.5	56.7	0.04
6	11/33	0/2	0/2	N/A	N/A	N/A	N/A
7	0101/0101	Not done	Not done	N/A	N/A	N/A	N/A
8	0201/68	0/2	1/2	0.32	0.35	0.18	0.52
9	0201/0301	2/2	2/2	0.06	0.54	0.16	0.21
10	0201/0301	1/2	2/2	0	0.11	0	0
11	0201/23	0/2^c^	0/2	0.03	0.33	0	0
12	0101/23	0/2	1/2	N/A	N/A	N/A	N/A

*TIL* tumor-infiltrating lymphocyte, *PBMC* peripheral blood mononuclear cell

^a^Of the TIL cultures used for REP: number of reactive TIL cultures/number of TIL cultures assayed

^b^% MART-1 was defined as the percentage of CD8 + cells binding HLA-A*02:01/MART-1 multimers. Only patients expressing HLA-A*02:01 were evaluated for MART-1/Melan-A specificity

^c^One TIL culture from Patient 11 exhibited IFN-γ activity, but it was not reduced in the presence of anti-HLA Class I antibody

The presence of various T-cell receptor (TCR) beta chain variable region (Vβ) domains in the infusion products, as well as in peripheral blood samples taken before and after TIL infusion, was analyzed using flow cytometry. To use Vβ repertoire as a surrogate indicator of engraftment of the TIL infusion product, we asked whether there was evidence of one or more dominant Vβ populations in the infusion product, and if so, whether the dominant population(s) could be found in blood after infusion. Eight patients had one or more dominant Vβ populations in the CD8^+^ compartment of their infusion product (Supplementary Table 1). Of these eight patients, two patients had the same Vβ populations at 1 and/or 4 weeks after infusion that were present in their infusion product. Four patients had the same Vβ populations at 1 week (but not at 4 weeks) after infusion that were present in their infusion product. In two patients, the Vβ population that was present in the infusion product was no longer dominant at 1 week or 4 weeks after infusion. The CD4^+^ compartments of the infusion products had far fewer dominant Vβ populations compared to the CD8^+^ compartments (Supplementary Table 2).

Collectively, the Vβ flow cytometry data provide indirect evidence that the TIL product was capable of engrafting. Notably, one patient (Patient 1) had a dominant Vβ13.1 population in the infusion product (52% of CD8^+^ T cells), and then also in blood at 1 week after infusion (76%) which was still present at high levels at time points long after infusion (Fig. 2d and Supplementary Fig. 2). Interestingly, this patient had a dominant Vβ16 population that was detected 4 weeks after TIL infusion and remained at high levels past 6 months post-infusion, despite the fact that Vβ16 was not enriched in the TIL infusion product (Supplementary Fig. 2). At week 65, we analyzed PD-1 expression on the Vβ13.1 population which was also dominant in the TIL infusion product. At this time point, over 20% of the peripheral CD8^+^ T cells expressed a Vβ13.1 TCR. We found that a higher percentage of these Vβ13.1^+^ cells were PD-1^+^ as compared to non-Vβ13.1-expressing peripheral CD3^+^ cells (Fig. 2e). Expression of PD-1 on CD8^+^ T cells in the peripheral circulation of melanoma patients has been linked to tumor specificity of these cells [22]. Together, these data provide evidence that supports the possibility that there were tumor-specific cells in the infusion product that persisted in a responding patient for more than a year after TIL therapy.

## Discussion

Here, we report on the results of a phase II trial of a modified TIL treatment protocol for patients with advanced melanoma, substituting low-dose, subcutaneous IL-2 for the standard high-dose intravenous administration. When response was assessed per RECIST v1.1 criteria, there were two confirmed PRs and one unconfirmed PR observed amongst the 12 patients treated. This rate of response met the pre-specified criteria to consider the treatment effective. Despite meeting the statistical threshold for efficacy, a caveat to our results was that no patient achieved a CR and none of the PRs were durable. It is possible that the low response rate compared to other published clinical trials of TIL therapy for metastatic melanoma was due to the low-dose IL-2 regimen in our protocol; randomized trials would be needed to address this. It was also noted that 3 of the 12 patients treated in our study had mucosal or ocular melanoma. The response rate to TIL therapy and immunotherapy appears to be lower in these subsets [23–25]. None of these three patients responded in our study; therefore, the response rate of patients with cutaneous melanoma was 22% (2/9).

The observed toxicity attributable to the IL-2 was acceptable, with the majority of adverse events being grade 1 or grade 2. These toxicities were managed in a non-ICU setting and no patients experienced broad cardiovascular collapse; this is in contrast to patients treated with high-dose IL-2 therapy. Toxicities generally could be reversed with supportive measures and/or by delaying or omitting subsequent IL-2 injections. The toxicity profile observed from the subcutaneous, low-dose IL-2 used in this trial is similar to the previous report of a TIL protocol that utilized a decrescendo intravenous dosing strategy [14]. Thus, low-dose IL-2 regimens may represent alternatives to high-dose IL-2 in future trials, especially when TIL therapy is combined with other immunotherapies such as immune checkpoint blockade.

Persistence of the transferred T cells has been strongly associated with response to TIL therapy [2, 14]. Therefore, a lack of persistence of the infused TIL is another possible explanation for the lack of durable responses observed in our study. However, in addition to the suggestion of persistence of the transferred cells in Patient 1 (Fig. 2d), we also observed enrichment of MART-1/Melan-A-specific T cells in some of the non-responding patients, particularly Patients 4 and 9, a month after TIL transfer (Table 4). Recent data from Donia et al. have suggested that the persistence of the transferred cells alone may not be sufficient to provide benefit from TIL therapy and that tumor regression requires the accumulation of polyfunctional, PD-1^+^ T cells in the peripheral circulation after TIL infusion [26]. This observation is in accordance with our observation of high PD-1 expression on the Vβ13.1^+^ T cells in Patient 1 (a responder) (Fig. 2e). It also supports an emerging paradigm that these peripheral PD-1^+^ T cells target tumor neo-antigens and that response to ACT correlates with the mutational burden of the tumor and the recognition of tumor neo-antigens, as opposed to shared tumor-associated antigens, by the transferred TIL [22, 27, 28]. Tumor neo-antigen load and mutational burden have also been correlated with response to anti-CTLA-4 therapy and overall survival with anti-PD-1 treatment in patients with melanoma [29–31]. Given that 11 of the 12 patients treated in our trial had previously progressed on at least one line of immune checkpoint inhibitor therapy (Table 1), it is possible that our cohort included patients, whose tumors possessed relatively low neo-antigen loads. Studies are ongoing to further test this hypothesis by elucidating the antigen specificity of the transferred TIL as well as the mutational landscape of the patients’ tumors.

If PD-1^+^ CD8^+^ T cells targeting tumor neo-antigens are indeed the major mediators of response to TIL therapy, this would also suggest that these cells are susceptible to regulation via the PD-1/PD-L1 axis. Interestingly, both the patients with confirmed PRs on this study, Patients 1 and 8, subsequently went on to have durable responses to anti-PD-1 therapy after progression on TIL therapy. This was particularly notable for Patient 8 as his disease had progressed previously on anti-PD-1 therapy before TIL treatment, suggesting a potential synergy between the transferred TIL and the subsequent anti-PD-1 therapy. In addition, a dense infiltrate of PD-1^+^ CD8^+^ T cells was observed in a biopsy from a progressing cutaneous lesion post-TIL therapy in the patient with a PRu by RECIST (Patient 7) (Supplementary Fig. 3). It is tempting to speculate that treatment with anti-PD-1 could have re-invigorated these infiltrating cells and led to prolonged tumor regression.

Our experience with this small cohort of patients supports the rationale for future studies that combine TIL therapy with subsequent anti-PD-1/PD-L1 treatment. Currently, we are accruing patients who have progressed on previous anti-PD-1/PD-L1 therapy to a trial of ACT that incorporates both low dose, subcutaneous IL-2 and anti-PD-1 therapy after TIL infusion (NCT03158935). The goal of this trial is to improve the depth and durability of responses to our ACT regimen whilst still limiting the treatment-related toxicities of IL-2.

### Electronic supplementary material

Below is the link to the electronic supplementary material.

Supplementary material 1 (PDF 2199 KB)

## Data Availability

The portions of the datasets generated or analyzed during the current study that are contained within confidential patient records are not publicly available, but the data that support the findings of this study that do not contain any identifiable information are available on reasonable request from the corresponding author.

## Abbreviations

AJCC: American Joint Committee on Cancer
CR: Complete response
CTCAE: Common terminology criteria for adverse events
ECOG: Eastern Cooperative Oncology Group
G-CSF: Granulocyte colony-stimulating factor
irRC: Immune-Related Response Criteria
MART-1: Melanoma antigen recognized by T cells 1
PD: Progressive disease
PR: Partial response
PRu: Unconfirmed partial response
REP: Rapid expansion protocol
SD: Stable disease
