# Supplementary material for: Phase II clinical trial of adoptive cell therapy for patients with metastatic melanoma with autologous tumor-infiltrating lymphocytes and low-dose interleukin-2
Source: Cancer Immunol Immunother. 2019 Feb 11;68(5):773–85. doi: 10.1007/s00262-019-02307-x (PMC11028227; doi:10.1007/s00262-019-02307-x)
Supplement: Supplementary file 1 — Supplementary material 1 (PDF 2199 KB) [file 262_2019_2307_MOESM1_ESM.pdf]

Patient	Infusion Product	Week 1 post-infusion	Week 4 post-infusion
1	Vβ13.1 (52%)<br>Vβ8 (21%)	**Vβ13.1 (76%)***<br>**Vβ8 (8.9%)***<br>Vβ16 (4.2%)	
2	Vβ8 (14%)		
3			
4		Vβ13.1 (20%)	Vβ13.2 (36%)<br>Vβ23 (19%)
5	Vβ13.2 (54%)<br>Vβ17 (7.8%)<br>Vβ2 (26%)	**Vβ13.2 (56%)***<br>**Vβ17 (19%)***<br>**Vβ2 (7.0%)***	
6		Vβ5.1 (16%)<br>Vβ22 (18%)	Vβ17 (13%)<br>Vβ2 (12%)
7	Vβ5.1 (38%)	**Vβ5.1 (17%)***	
8			
9	Vβ2 (13%)	Vβ14 (11%)	Vβ21.3 (16%)
10	Vβ17 (12%)<br>Vβ5.1 (9.8%)<br>Vβ1 (11%)	**Vβ17 (87%)***<br>**Vβ5.1 (12%)***	**Vβ17 (11%)***
11	Vβ7.1 (92%)	**Vβ7.1 (87%)**	Vβ2 (11%)
12	Vβ13.1 (15%)<br>Vβ22 (41%)	**Vβ13.1 (25%)***<br>Vβ2 (12%)	Vβ2 (10%)<br>**Vβ22 (15%)***

Vβ, T cell receptor beta chain

***Bold** denotes Vβ population that was also dominant in the infusion product

Supplementary Table 1. Dominant TCR Vβ populations in the CD8+ compartment

The TCR Vβ repertoire of the $CD8^+$ compartment of TIL infusion products, and peripheral blood at 1 and 4 weeks post-infusion were analyzed using the IOTest Beta Mark Kit (Beckman-Coulter). Shown are the dominant TCR Vβ populations, as defined by any Vβ chain whose frequency was considered to be a statistical outlier in the repertoire of the 24 Vβ chains that were analyzed. An outlier test was used to define a Vβ as dominant if its frequency was at least three interquartile distances away from the third quartile of all the Vβ chains analyzed.

Patient	Infusion Product	Week 1 post-infusion	Week 4 post-infusion
**1**		Vβ5.1 (10%)<br>Vβ2 (12%)	
**2**			Vβ2 (17%)
**3**			
**4**	Vβ2 (16%)		
**5**	 Vβ2 (18%)	Vβ17 (30%)<br>**Vβ2 (16%)***	
**6**		Vβ17 (8.3%)	 Vβ2 (11%)
**7**			
**8**		Vβ4 (23%)	Vβ4 (55%)
**9**	Vβ23 (12%)<br>Vβ8 (12%)		
**10**			
**11**			Vβ2 (11%)
**12**			Vβ2 (11%)<br>Vβ17 (8.9%)

Vβ, T cell receptor beta chain

***Bold** denotes Vβ population that was also dominant in the infusion product

Supplementary Table 2. Dominant TCR Vβ populations in the CD4+ compartment

The TCR Vβ repertoire of the CD4+ compartment of TIL infusion products, and peripheral blood at 1 and 4 weeks post-infusion were analyzed using the IOTest Beta Mark Kit (Beckman-Coulter). Shown are the dominant TCR Vβ populations, as defined by any Vβ chain whose frequency was considered to be a statistical outlier in the repertoire of the 24 Vβ chains that were analyzed. An outlier test was used to define a Vβ as dominant if its frequency was at least three interquartile distances away from the third quartile of all the Vβ chains analyzed.

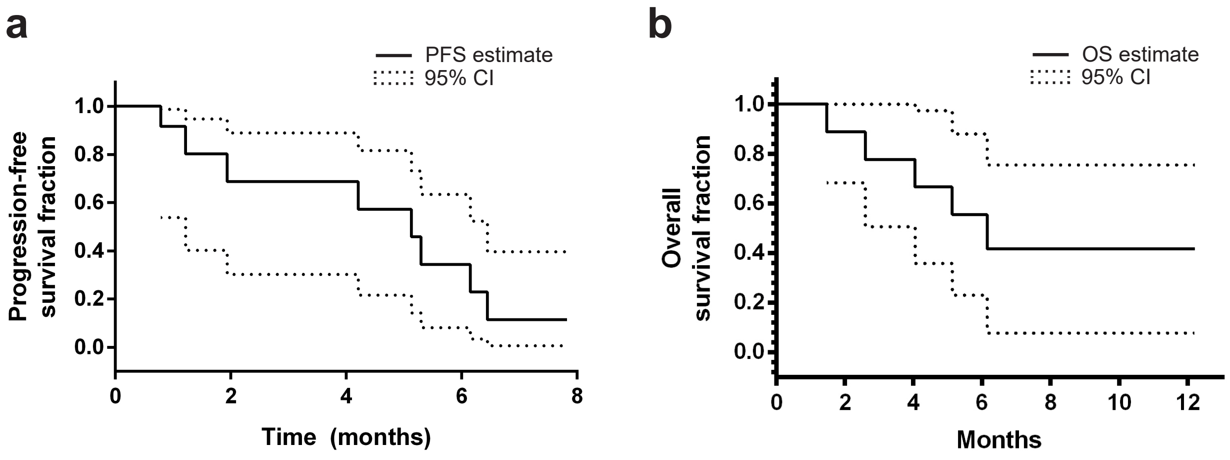

Supplementary Figure 1. Survival curves

a. Progression-free survival. Twelve patients were included in the analysis and eight events (both RECIST and irRC PD or death) were observed. The estimated median PFS time was 5.1 months (95% CI: 1.2 – 6.4 months) **b.** Overall survival. Twelve patients were included in the analysis and five events (death) were observed. Median OS was estimated to be 6.2 months (95% CI: 1.5 to not reached).

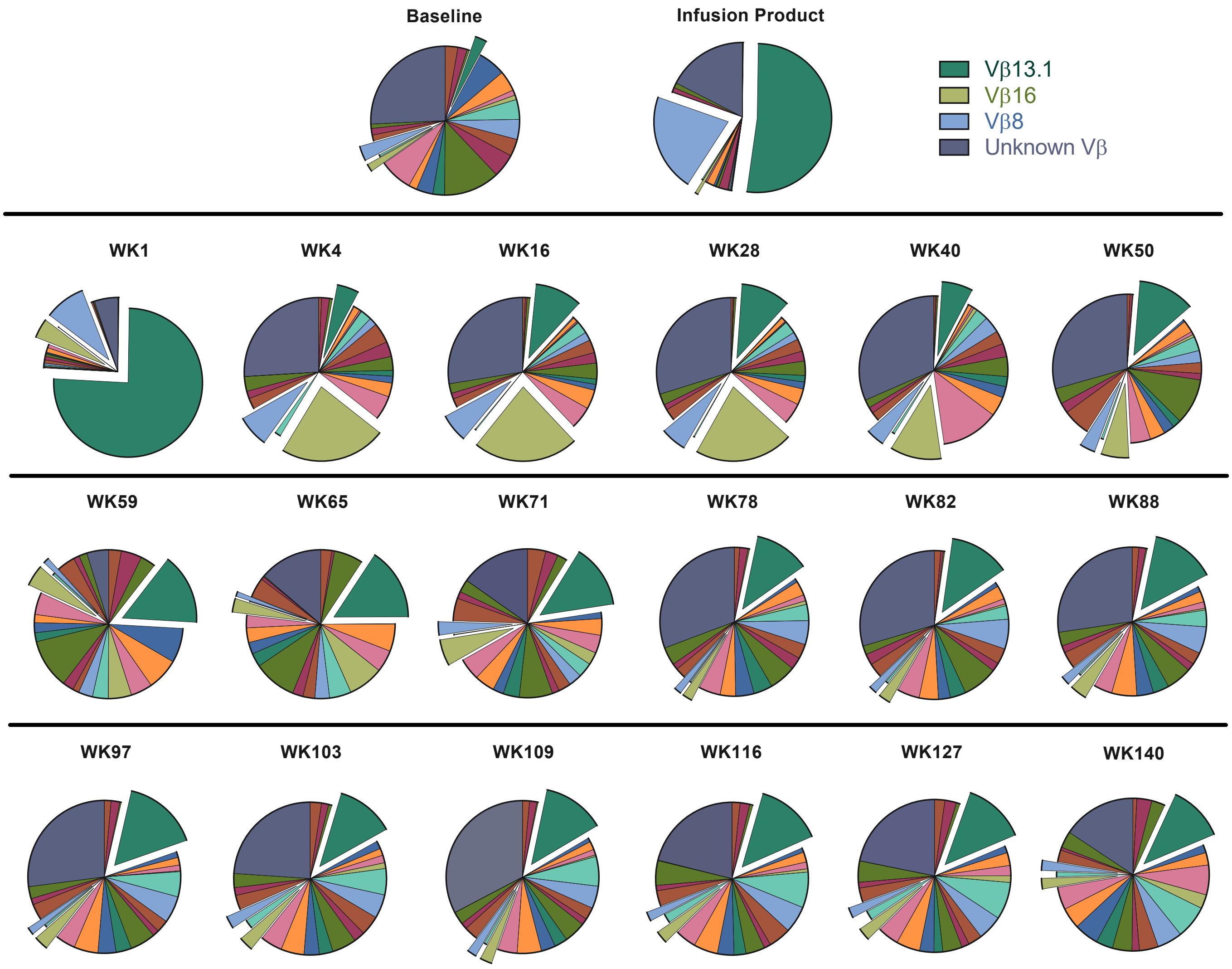

Supplementary Figure 2. Vβ repertoire analysis of CD8+ compartment (Patient 1)

Peripheral blood mononuclear cells taken before TIL therapy (Baseline) and after TIL infusion (WK1 – WK140) were analyzed by flow cytometry for the proportion of various TCR Vβ chains present in the CD8+ T cell compartment. This analysis was also performed on a sample of the TIL infusion product. The legend describes the color coding for three TCR Vβ populations of interest that are exploded from the pie charts: Vβ13.1, which was dominant in the infusion product and at many time points post-infusion; Vβ16, which was not dominant in the infusion product but expanded in peripheral blood post-infusion; and Vβ8, which was dominant in the infusion product and then declined post-infusion. The legend also indicates the population of T cells expressing TCR Vβ chains that were not interrogated by the Vβ antibody panel used (unknown Vβ chains).

Patient 7 - Week 17 post-treatment biopsy

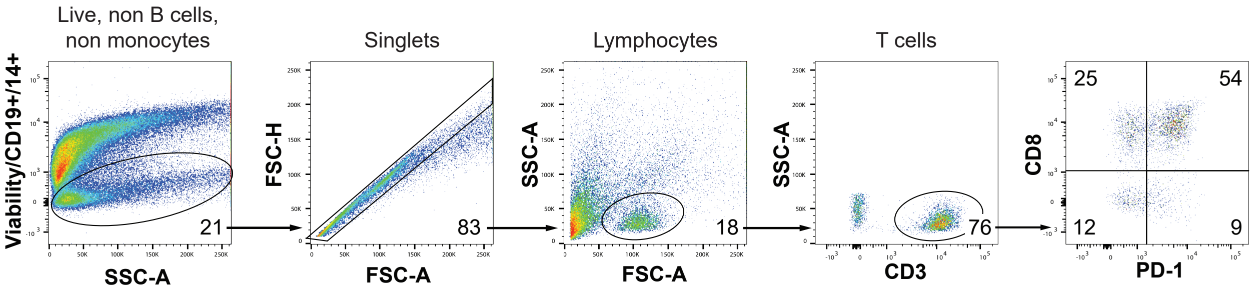

Supplementary Figure 3. Flow cytometric analysis of post-treatment biopsy (Patient 7).

A subcutaneous lesion was surgically removed from Patient 7 at 17 weeks following TIL infusion. After enzymatic dissociation of the tissue, the above gating strategy was applied to identify CD3+ lymphocytes for analysis of CD8 and PD-1 expression by flow cytometry.
